# The Modified Caldwell-Luc Approach with the Use of Collagen Material for Treatment of a Chronic Perforated Maxillary Sinusitis

**DOI:** 10.1155/2020/8874227

**Published:** 2020-10-27

**Authors:** Ekaterina Diachkova, Elena Morozova, Natalia Blagushina, Svetlana Tarasenko

**Affiliations:** Department of Dental Surgery of Borovsky Institute of Dentistry, Sechenov First Moscow State Medical University (Sechenov University), Russia

## Abstract

Oroantral fistula (OAF) often develops after extraction of posterior maxillary teeth and requires surgical closure. If it persists, OAF may result in maxillary sinusitis. This paper reports a case of an oroantral fistula, associated with chronic maxillary sinusitis. A 46-year-old female patient presented with a history of traumatic tooth extraction, which led to OAF formation. Three unsuccessful attempts were made to close it elsewhere. With one operation, we performed Caldwell-Luc surgery and closed OAF with a collagen membrane and plug and a buccal flap. The patient was assessed at 1-, 3-, 6-, and 12-month and 8-year follow-up visits, with no signs of maxillary sinusitis or OAF recurrence being found with the efficient amount of bone and opportunity for further dental rehabilitation like sinus lifting and dental implantation. We believe that this approach may be a viable option in similar cases.

## 1. Introduction

Oroantral fistula (OAF) is a relatively frequent complication after extraction of posterior maxillary teeth [1]. Therefore, surgeons should be alert to the possibility of its formation, especially when the first and/or the second upper molars are to be extracted [2]. Nevertheless, even gentle extraction does not always guarantee against OAF formation, so a surgeon should be capable of treating this complication.

Immediate postextraction oroantral communications must be closed within 48 hours following surgery. If primary closure has not been achieved within these time limits, the internal surface becomes epithelised and a chronic OAF develops. In such cases, spontaneous closure is impossible [3]. The established communication of the maxillary sinus with the oral cavity is likely to lead to chronic odontogenic maxillary sinusitis. This condition requires a combined approach as treatment of either OAF or sinusitis alone is insufficient [4]. Also, there are no articles that described the treatment of OAF with the maxillary sinusitis when besides the sanitation and covering of communication, authors provided the techniques for stimulation of osteogenesis and creation of good condition for further dental rehabilitation.

In the present case report, we describe a surgical protocol for the treatment of OAF associated with chronic maxillary sinusitis.

## 2. Clinical Case

A 46-year-old female patient was referred to the Oral and Maxillofacial Surgery Clinic (Sechenov University, Moscow, Russia) with oroantral fistula, associated with left-sided chronic maxillary sinusitis. The patient reported that tooth 2.6 had been extracted elsewhere three years before and afterwards, she noticed leakage of liquids through the nose while drinking. The patient visited a dental surgeon where she was diagnosed with oroantral fistula (OAF). However, an attempt to close it failed and thereafter so did two other attempts. The patient also complained of recurrent episodes of facial pain and pressure.

On examination, OAF and scar tissue at the postextraction site were observed in the projection of absent tooth 2.6. Besides that, tooth 2.5 was found to be extensively damaged so it could not be restored. The surgical plan included extraction of tooth 2.5, maxillary sinusotomy, and OAF closure with a collagen membrane in one operation. The patient signed the informed consent form for operation and the use of results of treatment for scientific and educational purposes. The patient underwent full preoperative examination (laboratory blood test, biochemical blood test, chest X-ray, coagulogram, and other prescribing for preoperative examination before planned surgery), with no relevant clinical data being found. It was provided orthopantomography for additional investigation and evaluation of the condition of the maxillary sinus.

Surgical treatment included Caldwell-Luc sinusotomy and sinuplasty with the local tissues with our changes: for covering the bone window and oroantral connection and plugging the fistula, we used different forms of bovine collagen (“Collost,” BioFarmaHolding, Moscow, Russia) with the 3D orientation of fibrils that can increase the osteoconduction features of material.

**Figure 1:**
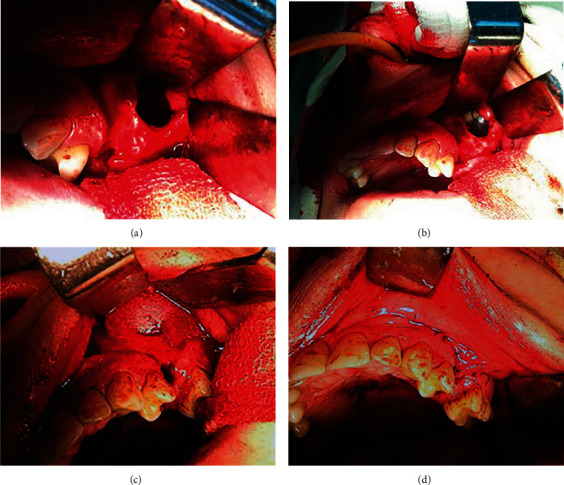
Surgical procedure: (a) sinus debridement through the created window; (b) insertion of the Foley catheter balloon into the sinus; (c) membrane placement over the window and the bony defect; (d) flap placement and suturing.

Local anaesthesia was obtained with 4% articaine hydrochloride with 1 : 100,000 epinephrine. A 5 cm semilunar incision encompassing OAF was made, and a full-thickness buccal flap was elevated. The oral mucosa adjacent to OAF was excised. Tooth 2.5 was extracted. A round window in the lateral wall of the maxillary sinus was prepared with a cylindrical diamond bur. Special care was taken to leave at least 15 mm of bone between the OAF and the window. The normal mucous membrane was left intact, whereas the diseased sinus membrane was debrided with curettes (Figure 1(a)). The internal surface of the sinus was thoroughly irrigated with 0.05% chlorhexidine gluconate. An inferior meatal nasal antral window was created with a chisel and widened with a rongeur. A deflated balloon of the Foley catheter was placed into the sinus, and a tube was passed through the nasal antral window to the outside.

The balloon was then inflated with 10 ml of sterile saline (Figure 1(b)). The flap was mobilised to ensure full coverage without tension. The extraction socket of tooth 2.5 was filled with a cross-linked bovine collagen matrix. Both the oroantral window and OAF were covered with the collagen membrane (Figure 1(c)). The flap was repositioned and closed with 4/0 polypropylene sutures (Figure 1(d)). The surgical wound was covered with a previously made protective silicon plate, and the patient was instructed to use it when eating.

The patient was prescribed with ceftriaxone 1 g IM twice daily for 7 days, loratadine 10 mg once daily for 7 days, ibuprofen 600 mg thrice daily for 4 days, and oxymetazoline 0.05% nasal spray twice daily for 7 days.

The Foley catheter and the sutures were removed 1 and 7 days after surgery, respectively. No early postoperative complications occurred.

**Figure 2:**
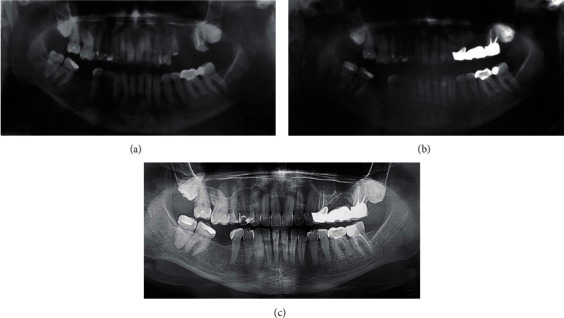
Panoramic radiograph: (a) preoperative radiograph showing bony defect at the postextraction site of tooth 2.6 and failing tooth 2.5; (b) radiograph taken 3 months after surgery showing bone repair; (c) radiograph taken at 8-year follow-up showing the stability of bone volume and absence of any pathological lesions.

The patient was assessed at 1-, 3-, 6-, and 12-month and 8-year follow-up visits. No signs of chronic maxillary sinusitis or OAF were found during clinical examination and orthopantomography. A significant increase in volume and density of bone at the surgical site is shown in a series of consecutive radiographs (Figure 2).

Two alternatives for further rehabilitation (i.e., dental implantation or placement of a fixed dental prosthesis) were offered to the patient. As the patient refused another surgical intervention, prosthodontic treatment was approved and provided at the fifth month after surgery. Both quality of life and working ability of the patient improved dramatically after completing the treatment.

## 3. Discussion

As a rule, OAF cannot be closed when sinus infection is present, so complete sinus debridement is mandatory before OAF closure. In this case, the patient required surgical treatment of maxillary sinusitis because the sinus membrane was irreversibly diseased and the patient reported of frequent acute exacerbations of maxillary sinusitis (up to 5 times a year), which occurred regardless of any previous attempts to treat this condition. To prevent reinfection, we decided to perform both sinus debridement and OAF closure in one operation.

To date, surgical techniques tend to be less invasive and radical. This is also true for maxillary sinus surgery, where the traditional Caldwell-Luc approach has been almost completely replaced by functional endoscopic sinus surgery. However, the latter requires high professional skills and special armamentarium, which is not always available. On the other hand, the Caldwell-Luc surgery is a simple, less expensive, and time-tested method, which yields good results [5]. Nevertheless, it is a relatively traumatic procedure, so we decided to place the Foley balloon into the sinus to minimise the risk of postoperative bleeding and swelling. Moreover, it made further membrane placement and suturing easier. Two retrospective studies of 55 cases reported good performance of the Caldwell-Luc surgery with balloon placement, with acute sinusitis being the only and rare complication [2, 6].

Several options for OAF closure are available, all of them having their advantages and disadvantages. The buccal advancement flap technique is the easiest way for closing both immediate and chronic OAFs. Despite some drawbacks, the vestibular depth reduction, and rather limited blood supply being among them, it yields good clinical outcomes [7]. To overcome these limitations, some authors suggested to use a buccal fat pad flap due to its better vascularization [8]. A palatal flap can be considered another option [9]. Nevertheless, a full-thickness flap alone may be insufficient, as the Schneiderian membrane may adhere to the oral mucosa, thereby hindering bone regeneration. This complication can be prevented by placing a collagen membrane to separate the maxillary sinus from the oral cavity. Moreover, some authors advocate placing an autogenous platelet-rich fibrin membrane over the collagen membrane to promote soft tissue healing. Although some promising results have been obtained, it is still unclear whether this approach has greater effectiveness than the collagen membrane used alone [10] or with the collagen plug that creates additional barrier between the oral cavity and maxillary sinus decreasing the migration of microorganism and preventing the recurrence of maxillary sinusitis and OAF.

To make dental implantation possible, bone grafting may be performed when residual bone volume is insufficient or a large bony defect is present. Different types of grafting materials (e.g., auto-, allo-, and xenografts) have been used for this procedure [4]. The bone plate harvested after creating a window in the lateral wall of the maxillary sinus can be used as an autogenous graft, which allows avoiding additional surgery and reducing total costs [11].

In some cases of OAF, simultaneous sinus floor elevation can make the surgical procedure easier as the area of the defect in the Schneiderian membrane decreases. This approach also facilitates further implant surgery. But it is contraindicated if any signs of sinus infection are present [12]. In our case, the patient had a 3-year history of chronic sinus disease and, furthermore, was unwilling to place a dental implant, so there was no special need for bone grafting. Therefore, this treatment modality was rejected.

Third molar autotransplantation has also been considered a promising treatment for OAF, which has many advantages, some of them being immediate restoring of a missed tooth, low cost, and possibility of orthodontic treatment. However, there is very scarce data of clinical performance in immediate postextraction oroantral communication closure and no data for chronic OAF closure [13]. So, we decided against this treatment in our case.

## 4. Conclusion

The use of bovine collagen membrane and plug for covering of area of oroantral fistula and bone window during the operation of sanitation of the maxillary sinus helped to get good postoperative results such as the absence of recurrence of inflammatory process (maxillary sinusitis) due to removing the connection with oral cavity, stopping the circulation of microorganisms, and persistent contamination of maxillary sinus, also, with the stimulation of regeneration of bone because of the element of osteoconduction in collagen material and creating the conditions for further dental rehabilitation.

## Data Availability

No data were used to support this study.
